# Progressive pseudorheumatoid dysplasia confirmed by whole-exon sequencing in a Chinese adult before corrective surgery

**DOI:** 10.1186/s13018-019-1061-9

**Published:** 2019-01-11

**Authors:** Yan Li, Yan Zeng, Zhongqiang Chen, Haisong Xin, Xiaoliang Li

**Affiliations:** 1 Department for Orthopedics, Peking University Third Hospital, Huayuanbei Rd 49#, Beijing, 100191 China; 2 Department for Orthopedics, People’s Hospital of Huanghua, Cangzhou, Hebei China

**Keywords:** Progressive pseudorheumatoid dysplasia, Whole-exon sequencing, Spinal surgery

## Abstract

**Background:**

Progressive pseudorheumatoid dysplasia (PPD) is a rare autosomal recessive skeletal dysplasia caused by mutations in the *Wnt1-inducible signaling pathway protein 3* (*WISP3*) gene. Available literatures in PPD emphasized treatment strategy for polyarthritis, while few mentioned spinal deformity and related surgical intervention.

**Methods:**

Here, we present a Chinese man with PPD who underwent spinal surgery twice because of canal stenosis and related symptoms caused by the disease. Whole-exon sequencing (WES) was performed to confirm diagnosis before the second surgery.

**Results:**

A homozygous missense mutation (c.395G>A/p.C132Y) in *WISP3* was identified that co-segregated with affected family members.

**Conclusions:**

Our study illustrated a surgical outcome of PPD and highlighted the significance of early diagnosis and individualized surgical strategy, and also verified the value of WES in the diagnosis of PPD.

## Background

Progressive pseudorheumatoid dysplasia (PPD) is an autosomal recessive disease characterized by spondyloepiphyseal dysplasia associated with progressive joint deformities, pain, stiffness, and swelling mainly in the spine and hip joints [1]. PPD is caused by mutation in the *Wnt1-inducible signaling pathway protein 3* (*WISP3*) gene, which consists of five exons and encodes a 354 amino acid protein [2]. *WISP3* belongs to the connective tissue growth factor (CCN) gene family, and the encoded protein plays an essential role in skeletal growth and cartilage homeostasis [3]. This disease is usually misdiagnosed as juvenile idiopathic arthritis or juvenile rheumatoid arthritis (JRA), and patients can receive many years of unnecessary treatment before correct diagnosis [4].

Whole-exon sequencing (WES) is useful for identifying rare monogenic inherited diseases [5], and the reducing cost of WES has improved the feasibility of its clinical use [6]. In this study, we employed WES to explore the potential causative genes in a Chinese PPD patient who underwent spinal surgical treatment twice.

## Methods

### Case presentation

**Fig. 1:**
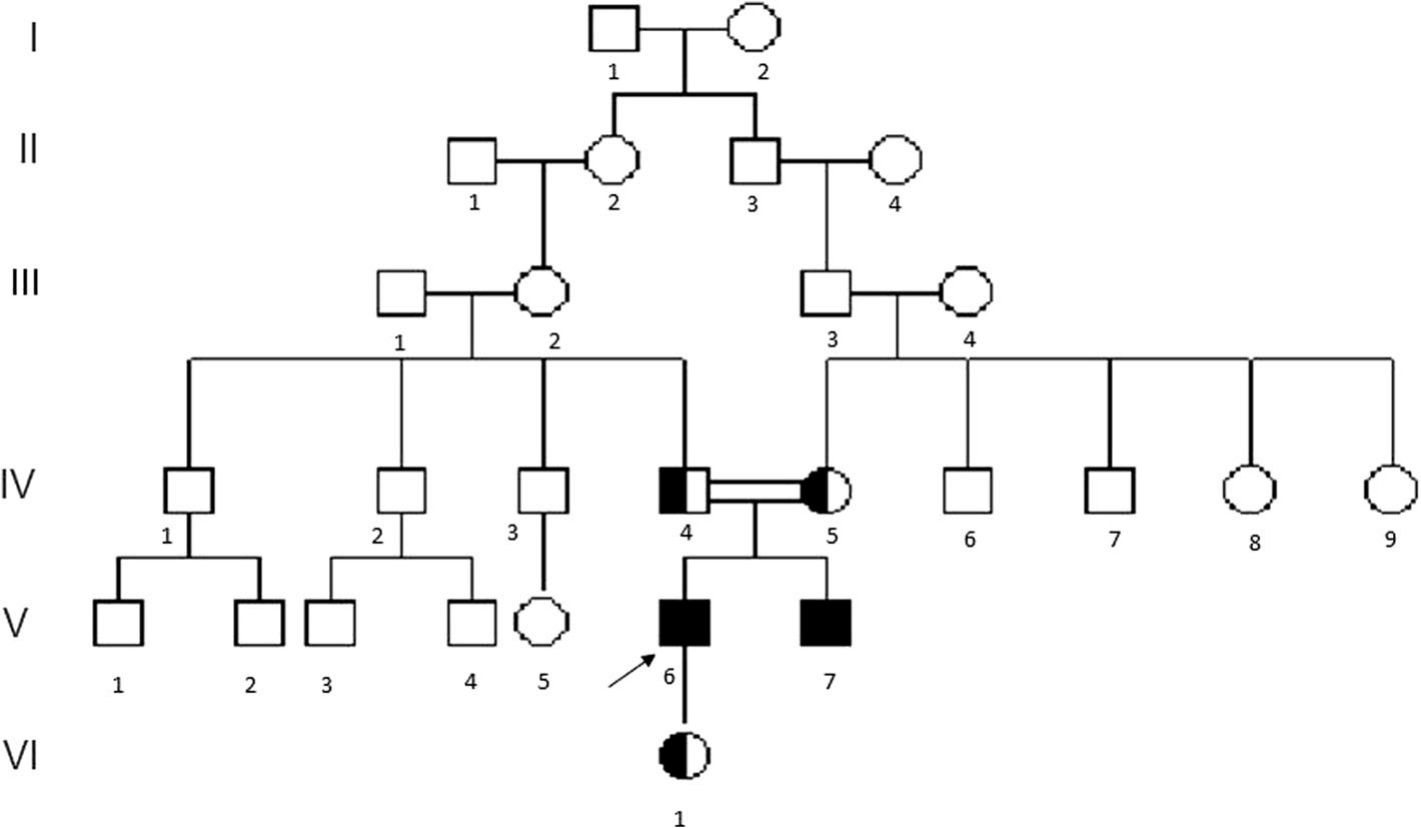
Pedigree of the studied Chinese family with progressive pseudorheumatoid dysplasia, presenting co-segregation of the c.395G>A mutation. The patient indicated by the arrow is the proband

**Fig. 2:**
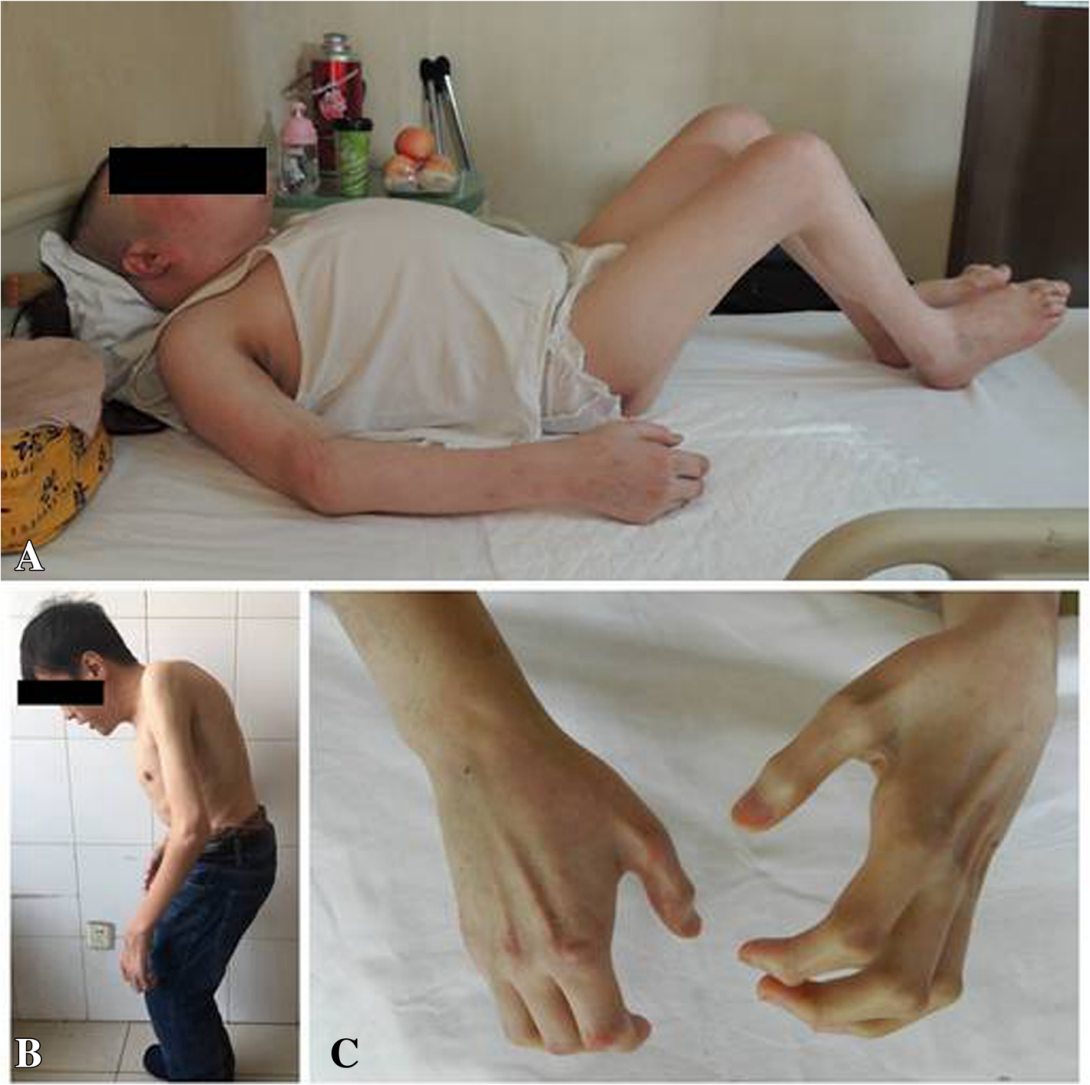
General views of the proband (**a**) and his younger brother (**b**) show kyphosis of the spine and multiple deformities of the extremities. The hands of the proband were initially involved, and their motion range was constrained by deformity (**c**)

The proband was a 35-year-old male from Hebei province in the north of China. He is of Han ethnicity and was born to consanguineous parents. His family pedigree is shown in Fig. 1. The proband was normally delivered after a full-term pregnancy, and birth weight and length were within normal ranges. The initial signs and symptoms appeared when he was 6 years old. Deformity of interphalangeal joints initially appeared in the fingers. Hips, knees, and wrists were then gradually involved. Diagnosis of JRA was considered by local hospitals, and glucocorticoids were prescribed without any efficacy. As he grew up, his symptoms deteriorated. He had to walk with crutches at 16 years of age because of arthritis of the lower extremities. At 26 years of age, he first experienced progressive pain with numbness radiating down his entire left leg and right thigh. At 34, he started to have mild difficulty in urination. Thereafter, his leg pain progressed and he became immobile. Treatment with tramadol, physical therapy, and spine injection were tried but were not effective. He had a younger brother with a similar clinical presentation but who also had mild neurological impairment (Fig. 2).

The proband’s height and weight were 162 cm and 72.5 kg when he was admitted to our hospital. His visual analogue scale (VAS) score was 9. He did not have behavioral difficulties and was not retarded in his intellectual development. Physical examination showed multiple malformations of the major limb joints, especially of the knees and hands (Fig. 2). Amyotrophy of both lower limbs was obvious. Cervical and lumbar movements were limited with compensatory kyphosis. The muscular strength of all four limbs was normal. Dysesthesia was found in the posterolateral left calf, dorsolateral left foot, and perineal area. Bilateral knee-jerk reflexes and ankle reflexes were hypo-induced. The erythrocyte sedimentation rate (13 mm/h) and C-reactive protein level (2 mg/L) were both within the normal range. Tests for rheumatoid factors were negative.

**Fig. 3:**
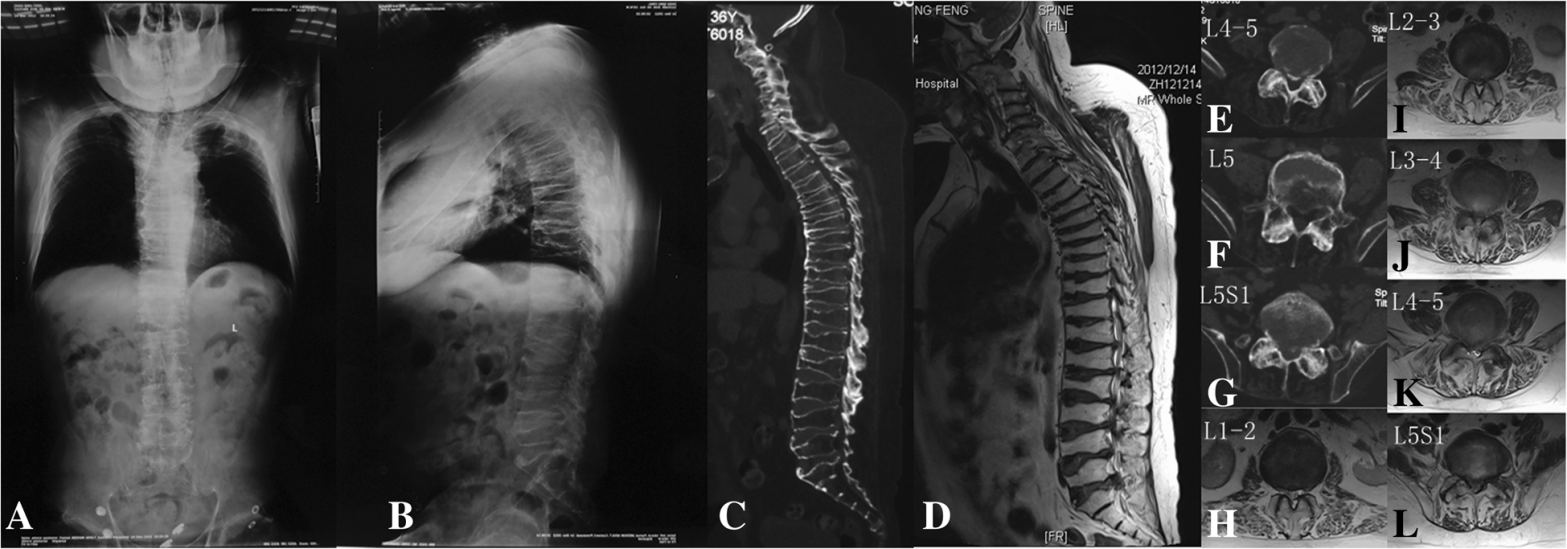
X-ray and CT scan of the whole spine showed osteoporotic flat vertebral bodies and bone bridges at multiple levels (**a**–**c**). Axial CT scan revealed maldevelopment of the left pedicle of L5 (**f**). Ossified herniated discs at L4–5 and L5–S1 were showed (**c**, **e**, **g**). Magnetic resonance imaging showed extensively canal stenosis from L2 to S1 (**d**, **h**–**l**)

Spinal x-rays showed flat and osteoporotic vertebral bodies. Pedicles were short, and end plates were irregular. Bone bridges were seen at many levels. Kyphosis was detected in both the cervical and upper thoracic spine. Magnetic resonance imaging showed multilevel Schmorl nodes. Multilevel disc herniation and hypertrophic ligamentum flavum caused lumbar canal stenosis from L2 to S1 (Fig. 3).

### DNA extraction

Blood samples were taken from the proband and from direct consanguineous relatives from three generations after an informed consent was obtained. DNA was extracted using a centrifuge column method (Tiangen, Beijing, China) and was qualitatively and quantitatively assessed by standard techniques before use in whole-exome sequencing.

### Whole-exon sequencing

All exons were captured using the Roche NimbleGen human exon V2 capture chip (Roche, Pleasanton, CA, USA) according to the manufacturer’s protocols, and sequencing data was obtained using the Roche NimbleGen human exon V2 capture chip on the Illumina HiSeq 2500 platform (Illumina Inc.).

**Fig. 4:**
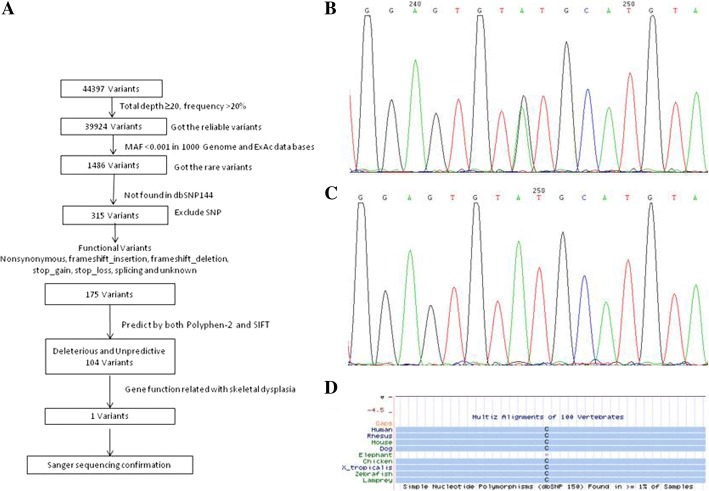
The data screening process of whole-exon sequencing revealed variants (**a**). Sanger sequencing chromatograms of family members who are heterozygous (**b**) and homozygous (**c**) for the *WISP3* variant. Amino acid conservation map across species demonstrating a highly conserved residue at position 132 (**d**, based on Multiz Alignments Track of UCSC Genome Browser and the human genome reference hg19/GRCch37)

The data filtering strategies were as follows: To identify reliable variations, those with a sequencing depth of at least 20 and a variation depth/total depth ratio higher than 20% were included. Because we were looking for rare mutations, those with minor allele frequency (MAF) higher than 0.1% in the 1000 Genomes Project and ExAc database were removed. Variants in the dbSNP 144 with MAF higher than 0.1% were also excluded. Synonymous and non-functional changes were also excluded. Deleterious variants located in the coding area of genes that were functionally related to skeletal dysplasia (Online Mendelian Inheritance in Man (OMIM), https://www.ncbi.nlm.nih.gov/omim) were selected using both PolyPhen-2 (http://genetics.bwh.harvard.edu/pph2/) and SIFT (http://sift.jcvi.org/). The procedure is described in Fig. 4.

### Segregation analysis

The putative mutation was validated by Sanger sequencing in the proband and four family members. The following PCR primers were designed using Primer 6.0 software (PREMIER Biosoft International, CA, USA): forward (CAGGGCACTGGACCATTAGA) and reverse (CCCACTGGTGCATGAAAACTAA). PCR products were purified and sequenced on an ABI 3130XL (Applied Biosystems). The results are shown in Fig. 4.

## Results

**Fig. 5:**
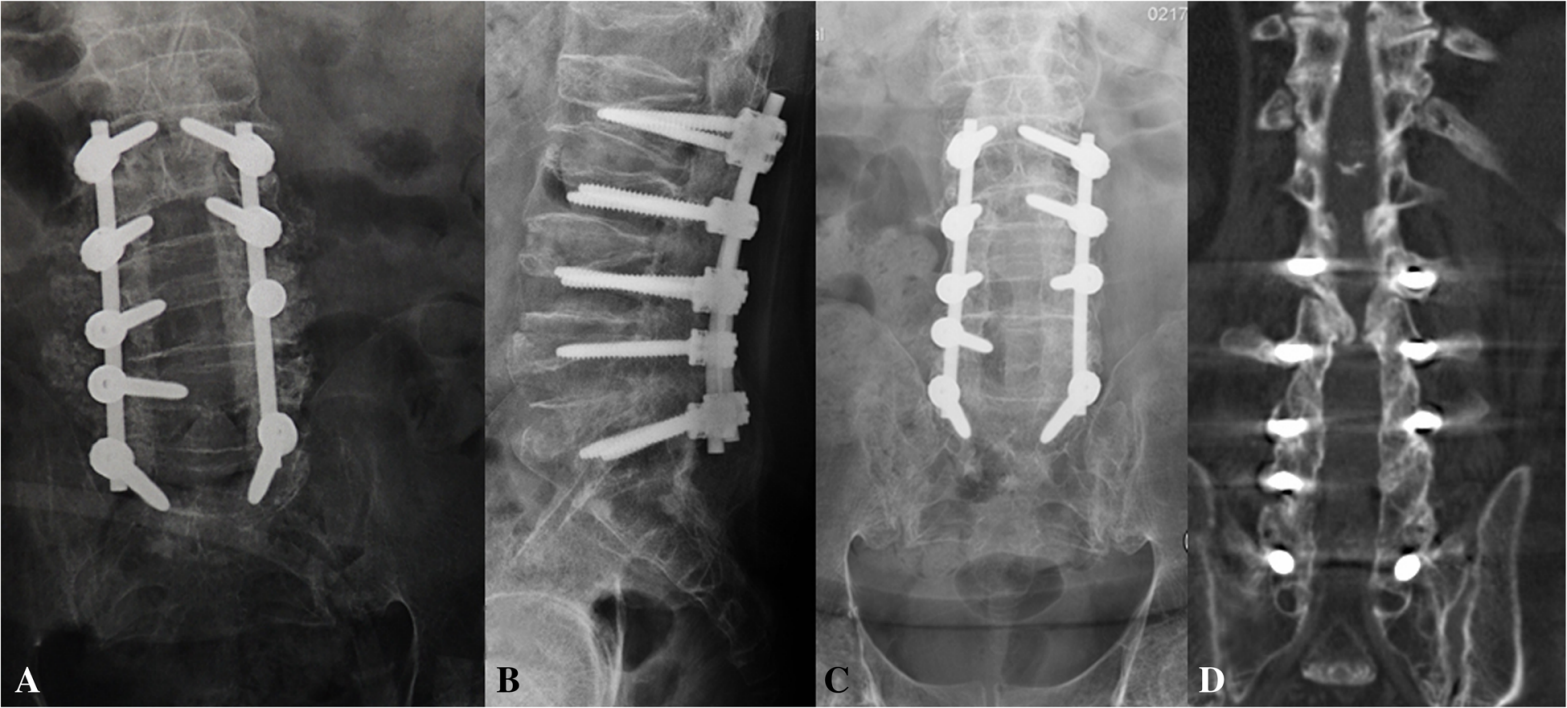
Extensive decompression of lumbar canal with internal fixation from L2 to S1 was implemented during the first surgery (**a**). X-ray and CT images suggest facet joint fusion 5 years later (**b**–**d**)

At the first admittance to our hospital, the diagnosis of the proband was not clear. Mucolipidosis type IV was suspected according to clinical presentations. Both physical examination and imaging indicated lumbar canal stenosis at that time, so we performed decompressive laminectomy and posterior-lateral fusion from L2 to S1. A pedicle screw internal fixation system was used (Synthes GmbH, USS II, Switzerland Inc.; Fig. 5), but the left pedicle of L5 was maldeveloped making it impossible to implant a screw (Fig. 3). Hypertrophic ligamentum flavum and herniated discs from L2 to S1 were confirmed during the surgery. The herniated discs of L4–5 and L5–S1 were ossified which caused cauda equina compression (Fig. 3). The dura at the L5–S1 level was hypertrophic and rigid. After the operation, leg pain was decreased and the VAS score was decreased to 1 without any surgical complications. With the help of rehabilitation training, the proband regained the ability to walk with the help of crutches. Bony fusion of the facet joints from L2 to S1 was achieved in the follow-up (Fig. 5).

**Fig. 6:**
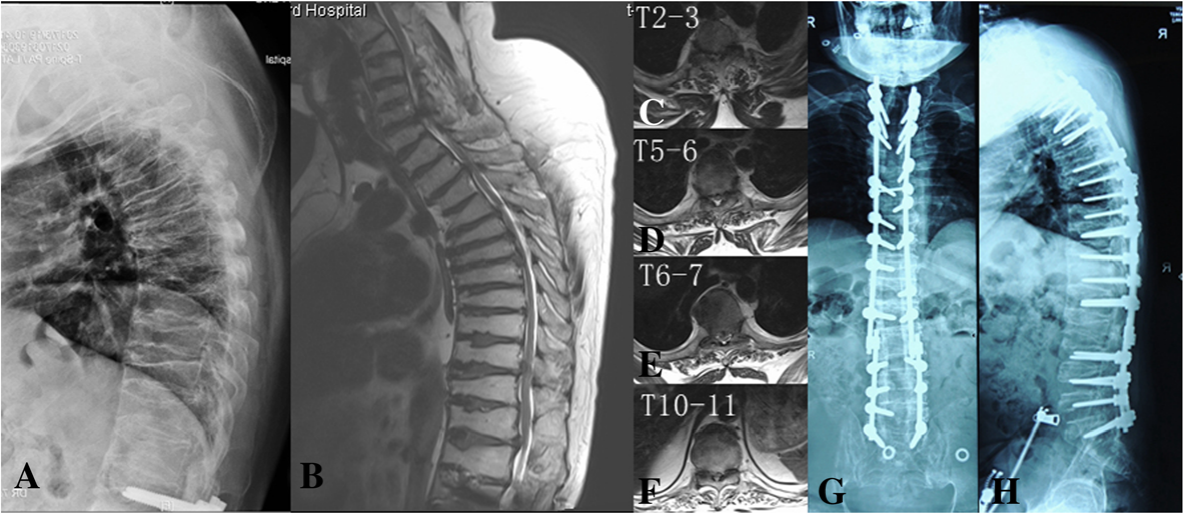
Before the second surgery, x-ray and magnetic resonance imaging of the thoracic spine showed kyphosis and multilevel canal stenosis (**a**–**f**). During the second surgery, we extensively decompressed the spinal canal from T2 to L2 and mildly corrected kyphosis of the middle thoracic spine (**g**, **h**)

Five years after the surgery, the patient began to feel stiffness and weakness of the lower extremities. His symptoms progressed for several months, and he lost ambulatory ability again. Physical examination revealed positive Babinski sign, hypesthesia below the xiphoid level, hypermyotonia, and extensive muscle weaknesses of the lower extremities. Magnetic resonance imaging showed extensive canal stenosis from T2 to L2 (Fig. 6). Kyphosis of the middle thoracic spine increased compression of the spinal cord, and Frankel classification was grade B. The proband underwent surgery again, during which decompressive laminectomy from T2 to L2 was performed and kyphosis correction was achieved by resecting the facet joints with circumferential decompression at the T5–6 level. We also removed the L2 pedicle screws and connected the segmental instrumentation (Synthes GmbH, USS II, Switzerland Inc.) with the previous internal fixation. Zonesthesia and hypermyotonia of the lower extremities improved postoperatively, but muscle weakness did not improve. At the latest follow-up, 1 year after the second surgery, the Frankel classification was still grade B.

Before the second surgery, WES was performed and a conclusive diagnosis of PPD was developed. WES covered 99.8% of target regions with a mean depth of on-target reads of × 105. A total of 44,397 raw variant calls were detected, and after removing variants with low quality (see the “Methods” section), 39,924 remained. Using the 1000 Genomes and ExAc databases, 1486 were selected as rare variants, 79.0% of which were excluded as known SNPs. Also excluded were 140 synonymous variants, while 71 benign or neutral variants were filtered out by PolyPhen-2 and SIFT. One rare variant (c.395G>A/p.C132Y) located in exon 3 of *WISP3* was identified as associated with skeletal dysplasia. Sanger sequencing confirmed this homozygous mutation co-segregated with affected family members (Fig. 4). This mutation resulted in the substitution of a cysteine with a tyrosine at position 132 and was predicted by Polyphen-2 as probably damaging with a score of 1.0 and damaging by SIFT with a score of 0. As shown in Fig. 4, this amino acid change affected a highly conserved residue.

## Discussion

PPD is a rare autosomal recessive disease, with an estimated population incidence in the UK of one per million [1]. In this study, we presented a family showing PPD and a patient undergoing spinal surgery twice. WES identified a missense mutation in *WISP3*, enabling a definitive diagnosis. We believe that this is the first presentation of a PPD patient receiving spinal surgery twice.

Patients with PPD are asymptomatic after birth and during early childhood, with symptoms often appearing between the age of 4 and 6 years [7, 8]. Because PPD patients are asymptomatic in the first years of life, the disease is usually misdiagnosed as mucolipidosis type IV and juvenile rheumatoid arthritis [9]. The typical clinical manifestations and radiographic findings in PPD include progressive deformities, pain, stiffness, and swelling of multiple joints, notably in the wrists, fingers, hips, and knees, with the absence of destructive bone changes [7]. To date, there is still no radical treatment for PPD and surgical treatment can be performed in individuals who might benefit. In our patient, the hypogenetic spine presented coin-like flat vertebral bodies, short pedicles, and kyphosis. These deformities resulted in developmental spinal canal stenosis. The degenerative changes, such as disc herniation and hypertrophic ligamentum flavum, worsened the spinal canal stenosis and gradually led to clinical symptoms. Spinal surgery aiming to decompress the spinal canal was inevitable if non-surgical intervention failed. Our patient also had developmental spinal canal stenosis and conspicuous spinal symptoms. He underwent lumbar spine and thoracic spine surgery successively. Available reports on the treatment for PPD focus more on hip joint replacement surgery, and this is the first reported sequential lumbar and thoracic spinal surgery. Meanwhile, vertebral body deformities, maldevelopment of the pedicles and segmental kyphosis, increased the difficulty of the spinal surgery. We noticed that muscle weakness did not improve after the second surgery, indicating the importance of early diagnosis and rehabilitation intervention.

*WISP3* belongs to the CCN family, which encodes multimodular mosaic proteins. To date, 51 mutations have been identified in *WISP3*, including missense, nonsense, frameshift, and exon-deletion mutations (the Human Gene Mutation Database (HGMD)), indicating that loss of *WISP3* function leads to PPD. Our study is consistent with another report of a Chinese patient with a similar phenotype and a *WISP3* p.C132Y mutation [10], which indicates this variant as a mutation hotpoint in the Chinese population. *WISP3* functions in the synthesis of chondrocyte saccharin and collagen type II [11], which are the major components of cartilaginous tissue. The loss of *WISP3* function can result in cartilage lesion.

WES enabled us to conclusively diagnose PPD in this patient and may provide wider clinical use in this field. With its decreasing cost, WES is increasingly used for diagnostic purposes in the clinical setting with a 25.2% diagnosis rate in a consecutive patient cohort [5]. With appropriate data filtering and pedigree mapping, WES can help develop diagnoses, especially in rare Mendelian diseases like PPD, which will help prevent the administration of unnecessary treatments [12]. Our study further verified the value of WES in the diagnosis of PPD. With the diagnosis of PPD, we were able to develop proper surgical strategy and anticipate complexity of surgery before the second procedure. Due to coin-like flat vertebral bodies of PPD, precisely applying pedicle screw should be pre-designed before surgery [13]. And since developmental spinal canal stenosis was induced by short pedicles in PPD, associated cervical, thoracic, and lumbar neurological symptoms may deteriorate over time with degenerative changes. As a result, spinal decompression range should be decided not only on the radiological imaging, but also on clinical manifestations. Favorable prognosis in PPD patients was also composed by patient education in early intervention of myelopathy and anti-osteoporosis medications.

## Conclusions

In conclusion, our study describes a PPD patient who underwent spinal surgery twice. Meanwhile, WES was used efficiently to identify causative mutations in PPD, thus informing a definitive diagnosis and enabling individualized surgical strategy.

## Abbreviations

HGMD: Human Gene Mutation Database
JRA: Juvenile rheumatoid arthritis
MAF: Minor allele frequency
OMIM: Online Mendelian Inheritance in Man
PPD: Progressive pseudorheumatoid dysplasia
VAS: Visual analogue scale
WES: Whole-exon sequencing
WISP3: Wnt1-inducible signaling pathway protein 3
